# Monitoring Soil Moisture Dynamics Using Electrical Resistivity Tomography under Homogeneous Field Conditions

**DOI:** 10.3390/s20185313

**Published:** 2020-09-17

**Authors:** Steven M. de Jong, Renée A. Heijenk, Wiebe Nijland, Mark van der Meijde

**Affiliations:** 1 Faculty of Geosciences, Utrecht University, 3508 TC Utrecht, The Netherlands; Renee.Heijenk@kcl.ac.uk (R.A.H.); W.Nijland@uu.nl (W.N.); 2 Department of Geography Strand, King’s College London, London WC2R 2LS, UK; 3 Department of Earth System Analysis, Faculty of Geo-Information Science and Earth Observation (ITC), University of Twente, 7500 AA Enschede, The Netherlands; M.vanderMeijde@Utwente.nl

**Keywords:** time-lapse ERT, soil water content, surface water, depth profile, infiltration, precipitation, spatial dynamics

## Abstract

There is a gap between lab experiments where resistivity–soil moisture relations are generally very good and field studies in complex environmental settings where relations are always less good and complicated by many factors. An experiment was designed where environmental settings are more controlled, the best outside laboratory, to assess the transferability from lab to outdoor. A field experiment was carried out to evaluate the use of electric resistivity tomography (ERT) for monitoring soil moisture dynamics over a period of 67 days. A homogeneous site in the central part of The Netherlands was selected consisting of grass pasture on an aeolian sand soil profile. ERT values were correlated to gravimetric soil moisture samples for five depths at three different dates. Correlations ranged from 0.43 to 0.73 and were best for a soil depth of 90 cm. Resistivity patterns over time (time-lapse ERT) were analyzed and related to rainfall events where rainfall infiltration patterns could be identified. Duplicate ERT measurements showed that the noise level of the instrument and measurements is low and generally below 3% for the soil profile below the mixed layer but above the groundwater. Although the majority of the measured resistivity patterns could be well explained, some artefacts and dynamics were more difficult to clarify, even so in this homogeneous field situation. The presence of an oak tree with its root structure and a ditch with surface water with higher conductivity may have an impact on the resistivity pattern in the soil profile and over time. We conclude that ERT allows for detailed spatial measurement of local soil moisture dynamics resulting from precipitation although field experiments do not yield accuracies similar to laboratory experiments. ERT approaches are suitable for detailed spatial analyses where probe or sample-based methods are limited in reach or repeatability.

## 1. Introduction

Soil moisture availability is a key factor in plant growth and vegetation development, both in natural systems and agricultural or plantation settings. Soil moisture is furthermore included in the list of essential climate variables (ECV) under the Global Climate Observing System [1] making it important to understand the dynamic processes of soil water uptake and re-distribution. Monitoring water availability to vegetation is difficult because access to the soil profile to quantify moisture is restricted without disturbing the soil system. Common methods with sensors to determine soil moisture content are gravimetric methods [2], time domain reflectometry (TDR) [3], neutron probe [4], cosmic-ray neutron method [5], ground penetrating radar (GPR) [4,6] and electric resistivity tomography (ERT) detailed below. Gravimetric measurements require coring into the deeper soil layers for sampling which is laborious, disturbs the soil body, and results in point (not spatial) observations. Furthermore, coring or digging for gravimetric sampling is often not feasible in stony and rocky soils often encountered in semi-arid and arid areas. TDR is a less disturbing methodology but has a footprint of only a few centimeters, and thus requires the placement of multiple electrodes at different depths to obtain soil moisture profiles to capture moisture dynamics. The neutron method is non-invasive and works at various spatial scales but it is difficult to separate the signal of soil moisture from vegetation and the deeper underground. GPR is a promising method for local or regional soil moisture estimates but it is difficult to separate the signal of moisture from clay content and it is difficult to drive quantitative estimates of moisture content from the GPR signal. A good overview of the various methods is provided by [4]. In this paper we focus on electric resistivity tomography (ERT).

Electrical resistivity measurements of the soil along a surface transect are an alternative method to obtain information on sub-surface conditions, soil moisture content, and moisture dynamics [7,8,9,10]. The method does not require coring, has minimal disturbance to the soil body (non-destructive measurement method), yields information to a depth of several meters, and is repeatable. If the assumption is made that the resistivity effects of structural soil properties remain constant through time, then differences in resistivity between repeat measurements may be attributed to changes in soil moisture content. Regular measurements of soil electrical resistivity during the growing season and after precipitation events can therefore yield important information on moisture availability and vegetation water use over time.

Various studies using ERT for monitoring soil moisture under controlled circumstances in laboratory settings are reported in the literature with good correlations between ERT-values and soil moisture values and low degrees of noise while field studies show modest correlation and disturbance by environmental factors. The use of geoelectrical tomography to quantify water distribution of soil in pots with, and without, life plant roots under well-defined boundary conditions was studied by [11] and they concluded that ERT is a reliable method for quantification of static and dynamic of soil water content in the pot root zone. A controlled soil column, an undisturbed soil monolith in a lysimeter, to evaluate potential and limitations of ERT for three dimensional soil moisture changes was used and described by [12]. In this study they succeeded in in situ calibration of the ERT-measurements for converting bulk electrical conductivity to water content and their conclusion is that ERT proved to be a suitable technique for observing soil water dynamics at the decimetre scale and that ERT is a promising tool to unravel the relationship between soil redistribution and root water uptake.

Earlier studies have shown the application of electric resistivity tomography to assess moisture dynamics in rocky soils in Mediterranean southern France [13,14]. ERT showed that plants extracted water from depths of up to 6 m because the roots are penetrating deep into the soil in between the stones and even into the cracks of the underlying bedrock [13]. Quantification of the water content was possible but requires localized, lithology specific, function to be fit empirically between the electrical resistivity soil water content [14]. Similar results were found in other Mediterranean areas on shale substrate [7], and in a mixed agricultural and treed landscape on limestone soil [15] where strong spatial patterns of water abstraction were observed around the scattered trees. In agricultural cropland settings ERT has been used to measure spatial patterns and dynamics in soil moisture content [16,17,18], and the effects of root water uptake and water redistribution within the soil profile [19,20,21]. ERT was used by [22] on both the soil and tree trunks and observed a strong coupling between soil matrix water potential and sap flow, but also indicators of deeper water use by trees during the summer.

The usefulness of ERT to study soil water availability for plants in vineyards was investigated by [23]. They conducted field experiments in plots of 7 by 7 m in Burgundy vineyards over a period of two years, relating ERT measurements to TDR soil moisture measurements to plant stem water potentials. In spite of a number of problems they were able to detect, in a qualitative way, hotspots in the soil for water absorption related to plant water deficit. Variability of soil properties and quantitative methods remain an important issue for further study. Brillante et al. [24] present a study where ERT measurements in a vineyard are related to soil moisture but also to soil properties such as soil texture, gravel content, cation exchange capacity, CaCO3, pH, organic carbon, and nitrogen. They aim at developing pedotransfer functions making the ERT measurements more applicable and transferable to different soil types and to heterogeneous soils. In their conclusions they stress the difference between homogeneous and heterogeneous soil when fitting relations between electric resistivity and soil water volume and problems with stony or clay-rich soils.

A detailed overview of geophysical techniques for landslides studies is provided by [25]. ERT is described as a promising and useful technique to collect quantitative information within landslides, especially shallow landslides, on hydrological properties such as water content and water distribution and dynamics, and geotechnical parameters such as the failure plane can faults or cracks.

The use of ERT for soil moisture monitoring in contour hedgerow mono and intercropping systems in field experiments is described and evaluated by [20]. They were mainly interested in patterns of soil moisture and less in quantitative assessment of moisture availability. Their conclusions were that ERT illustrates the effect of cropping systems and water retrieval on soil moisture distribution, but that information on soil horizons would be beneficial to capture spatial patterns.

Studies presenting the potential of ERT to capture soil moisture patterns and volumes are widely available in literature as shown above, but many of these studies also mention problems of relating electric resistivity measurements with soil moisture relations e.g., [7,13,14,21,23,24,26,27]. From the literature it is apparent that there is discrepancy between laboratory experiments [11,12] studying the relation between ERT-measurements and soil moisture under very controlled conditions with very good results, and the always complex field situations where ERT soil moisture relations are modest or poor caused by a wide range of environmental conditions having an impact on this relation. To obtain a better insight in the ERT measurement procedure and sensor response of the ERT data to changing and dynamic moisture conditions we designed an experiment where environmental settings are more controlled (the best outside laboratory) than is possible in the heterogeneous soils of the previous in situ studies [13,14]. A test location was selected with a homogeneous soil profile, known ground water levels, and precisely measured precipitation, where we performed ERT measurements over a period of 67 days (14 October to 20 December 2016). This study aims to evaluate multi-temporal ERT as a method to assess the spatial and temporal distribution of soil moisture in relation to rainfall in a homogeneous soil profile. The next sections will describe the details of study site, the applied methods, and analyses the performance of ERT in with reference to precipitation patterns and concurrent gravimetric soil moisture samples.

## 2. Study Site

**Figure 1:**
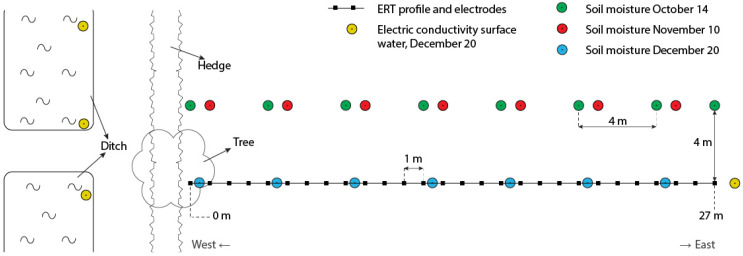
Plan view of the electric resistivity tomography (ERT) sensor 2016 set-up, including ERT electrodes, soil moisture measurement points, and surrounding features. The ERT transect of 27 m length is indicated by a black line and the small black squares indicate the positions of the electrodes with 1 m spacing. The colored circles illustrate the locations of the gravimetric sampling. October (green) and November (red) measurements were taken parallel to the ERT transect at a distance of 4 m away from the ERT transect to avoid disturbance to the ERT experiment. The December (blue) measurements were taken along the ERT transect in between the electrodes at the end of the experiment. Yellow circles represent the locations of Electric Conductivity (EC) measurements in surface or groundwater to determine possible effects on the ERT measurements.

**Figure 2:**
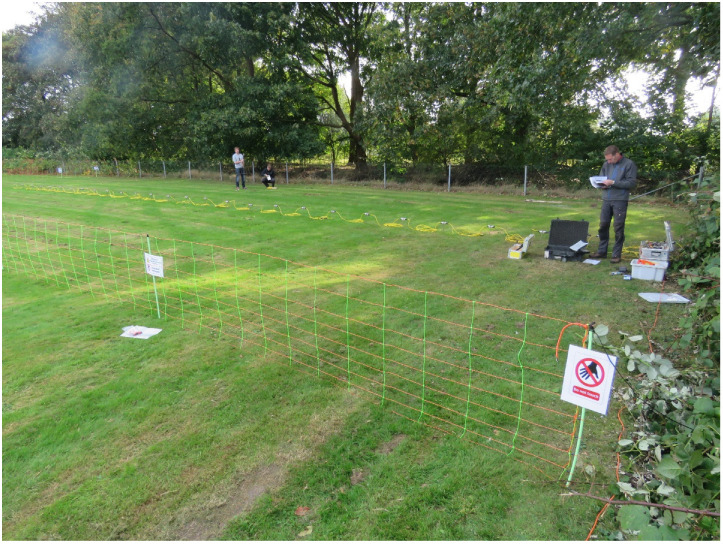
Experimental setup of the ERT experiment at the Royal Dutch Meteorological Institute (KNMI) in De Bilt. West is right in the picture, east is left (see also Figure 1). The picture is taken from approximately the word “Hedge” in Figure 1.

A test location for the ERT electrodes was selected at the Royal Dutch Meteorological Institute (KNMI) in De Bilt, in the center of The Netherlands located at (52.0987° N, 5.1756° E). The site is part of an open field housing a variety of atmospheric and meteorological measurement instruments including a continuous rain gauge. The KNMI test site was carefully selected and has a short, homogeneous grass cover where no management takes place other than mowing. No fertilizer is applied and consequently there is no pollution of the soil and groundwater that would interfere with ERT measurements. Two official auger points (BPK-143946 & 143972) of the Dutch Soil Survey are located close, within 200 m, and are available on: http://maps.bodemdata.nl/bodemdatanl/index.jsp. The upper 40 cm of the soil profile consists of a mixed clayey sand, with approximately 4% organic matter. The mixed layer sits on top of a homogeneous substrate of aeolian sands with a median diameter of the sand fraction of 155 µm. Groundwater (GW) level is relatively constant, controlled by the local water authority at approximately 180 cm below surface (Source: http://maps.bodemdata.nl/bodemdatanl/index.jsp). The ERT transect was positioned west to east perpendicular to a hedge. Figure 1 shows the position of the transect, a tree, the hedge, and a discontinuous ditch at the other site of the hedge. Figure 2 illustrates the experimental field setup.

## 3. Materials and Methods

### 3.1. ERT Setup

**Figure 3:**
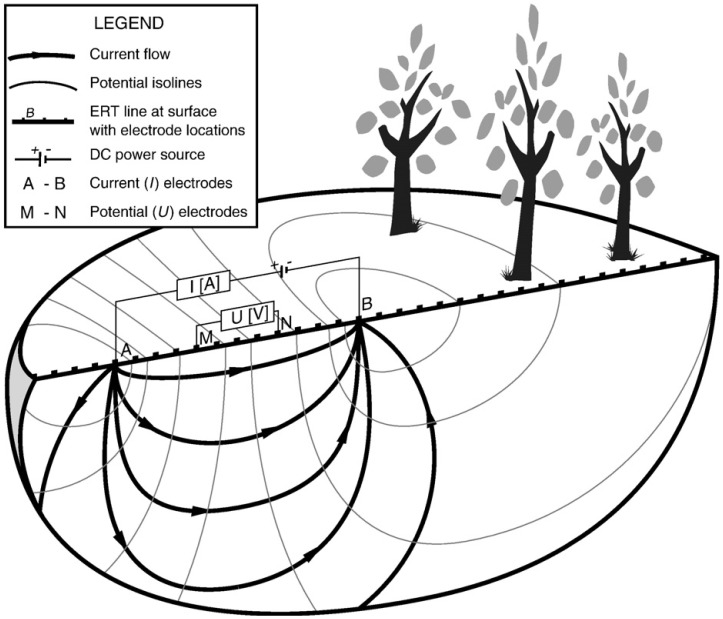
Schematic diagram of resistivity measurements in a homogeneous medium. By combining measurements of many electrode combinations using computer tomography, a spatial earth resistivity section is made and moisture dynamics can be captured [13].

ERT is a non-invasive technique to detect spatial and temporal variations in soil moisture and here applied to the KNMI experimental site. It provides information on the resistivity distribution of the subsurface which can be used for analyzing the composition and structure of the subsurface, but also to monitor dynamic processes over time, particularly fluid flow. To retrieve information on the subsurface conditions, a controlled direct electrical current is inserted into the ground via electrodes (Figure 3). At least four electrodes are needed: two to inject current into the ground, and two to measure the resulting potential difference. With an increasing electrode spacing deeper parts of the subsurface can be sampled. Combining measurements from many different electrode parings, with different spacing, along a transect results in a 2D resistivity section of the soil, which is dependent on lithology, porosity, structure, temperature, root density, and water content [28,29,30]. As mentioned before differences in measured soil resistivity over time is strongly related to changes in water content, but can also be influenced by temperature and pore fluid conductivity.

**Figure 4:**
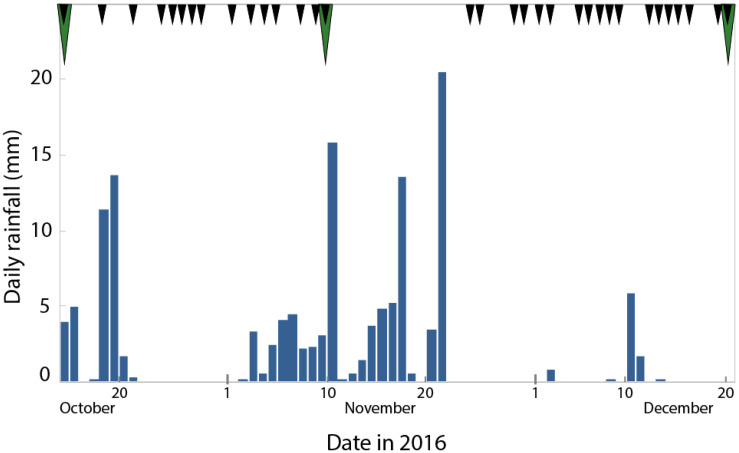
Daily rainfall measured at the experiment site and resistivity ERT measurement taken, the black triangles at the top of the image represent acquisition of ERT profiles. Gravimetric soil moisture was determined three times on 14 October, 10 November, and 20 December as indicated by the green triangles.

ERT data were collected using the Sting R1/IP advanced resistivity meter with a one-channel receiver with an array of 28 electrodes (www.agiusa.com). We used a Schlumberger configuration with 1 m spacing over a transect length of 27 m, which provides reliable resistivity information to a depth of approximately 5.5 m for the largest electrode spacings within the total line and provides the best trade-off between horizontal and vertical resolution [30,31,32,33]. We used a maximum “a” (distance between M and N, see Figure 3) of 3 electrode distances, and “na” (distance between A and M or B and N, see Figure 3) with ‘n’ between 1 and a maximum of 9. Data points with an elevated reciprocal and/or repeat error of 2% were removed in the acquisition process. Inversion of the data was done with EarthImager2D [30]. The inversion is based on a two-loop iterative nonlinear inversion. We solved the nonlinear inverse problem using the Gauss–Newton method with a calculated sensitivity matrix. The forward modeler that we used is based on a finite element method which produces more accurate forward modelling solutions than a finite difference approach, considering the same mesh discretization. We used a Cholesky decomposition forward solver which is numerically very robust and stable. Misfits are based on a standard misfit function and expressed in root mean square (RMS) or L2-norm. Inversions were stopped after four iterations or by a RMS error reduction of less than or equal to 1.5%. Our initial model is a homogenous half-space with the average apparent resistivity value as starting value. We use the same strategy for the absolute and time-lapse inversions with the difference that for follow-on steps in the time-lapse we use the reference measurement (first data take at 14 October) as starting point for the difference inversion (note that we do not subtract absolute value images but run an inversion on the differences, see for details [30,31,32,33,34]. The set-up was maintained on the site for 67 days and in total 33 ERT measurements were carried out (Figure 4). Gravimetric soil moisture was determined three times (Table 1).

A straightforward method to evaluate the stability of the ERT measurements is by stacking [34], a number of duplicate measurements are made apart from the automatic repeat measurements of the Sting R1/IP. Although more advanced methods to assess errors in ERT surveys exist, this approach provides a valuable field data quality assessment [35]. These duplicate measurements were carried out by immediately reiterating a measurement as soon as the previous survey was completed. The soil conditions and the moisture status were assumed not to have changed during these duplicate measurements and can so be used to assess internal instrument uncertainty for evaluation of the time-lapse inversion results.

Cumulative precipitation data are available at time intervals of 10 min on site as part of the KNMI meteorological observation network. Information on the site and instruments are available on the KNMI site (https://www.knmi.nl/research). We used daily cumulative values for analyses with ERT data.

### 3.2. Time-Lapse ERT Inversion

Time-lapse ERT inversion was done for two subsets, 14–24 October and 9–15 December, as two major rainfall events occurred during these time spans (Figure 4). Time-lapse inversion calculates the differences in resistivity profiles relative to the first measurement in each of the series, which uses the built-in time lapse inversion module of EarthImager2D [31]. The soil structure and lithological characteristics are assumed to remain constant during the experiment, and therefore all resistivity changes are attributed to soil moisture and temperature changes. Ground temperature at 5 cm below surface is recorded on site as part of the KNMI meteorological observation network (https://www.knmi.nl/research) and is evaluated here. According to Friedman [9] the effect of temperature is approximately 2% per 1 °C. In this case the difference in ground temperature is ±4 °C for the first period in October and ±1 °C for the second period in December resulting in 8% and 2% resistivity changes at the top 25 cm of the soil profile, respectively. Given the low variations of ground and air temperature during the experiment we assumed that at depths larger than 25 cm the temperature changes are negligible. Therefore, temperature differences were neglected in the analyses, but slight effects may be possible for the top of the soil profile in October.

### 3.3. Soil Moisture Sampling and Analysis

Soil samples have been taken at 7 or 8 locations (Figure 1) on three different moments through the ERT measuring period providing insight in soil moisture dynamics. Samples were taken at the beginning, middle, and at the end of the period (Table 1). On 14 October and 10 November, the samples were collected from boreholes at 4 m distance from the transect to avoid disturbance of the ERT measurements. On 20 December samples were taken on the ERT transect in between the electrodes (Figure 1). The samples were collected using an Edelman auger to drill a borehole. A steel Kopecky ring was then attached to the auger with a custom-made coupling piece. Samples were taken at around 12 cm, 40 cm, 60 cm, and 90 cm below the surface. The samples collected at the first date, 14 October, are more scattered in depth because of some problems with the sampling core and the explorative character of the first data collection. Soil samples were weighed, oven dried, and weighed again following the standard international procedure [2]. Gravimetric moisture content was calculated as

(1) $$\theta_{g}=\frac{M_{wet}-M_{dry}}{M_{dry}}$$

where *θg* is gravimetric moisture content, *M_wet_* is fresh sample weight (kg), and *M_dry_* is oven dried sample weight (kg).

### 3.4. Water Conductivity Analysis

At the end of the experiment the conductivity of the surface water (SW) to the west of the experiment site and of the groundwater at the east side of the experiment was measured using an electrical conductivity (EC) probe (Figure 1). The EC of the groundwater was 251 μS/cm at 3 °C and 241 μS/cm at 20 °C. Surface water had an EC of SW 720 μS/cm at 3 °C. The EC values were not calibrated for temperature, because only relative differences between values are used in this paper.

## 4. Results

**Figure 5:**
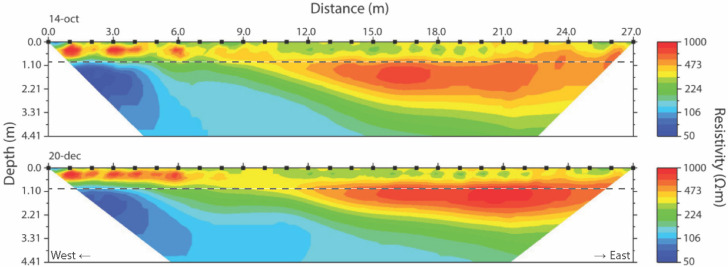
Resistivity profiles for the beginning (14 October) and the end (20 December) of the experiment along the 28 m transect. With inversion RMS-errors (%) of 1.20 and 1.13, respectively. The dashed line at 90 cm depth refers to the correlation analysis discussed further on.

Resistivity values in all the measured ERT profiles are between 50 and 1000 Ω-m. No obvious correlation is apparent between the resistivity profiles (Figure 5) and the geology sampled in the official Dutch Soil Survey BPK-boreholes at 200 m distance. The top 55 cm in both profiles deviates in resistivity in general above 500 Ω-m from the values found further down with values of below 500 Ω-m. The resistivity of the top layer is not uniform across the ERT transect and ranges from 200 to 600 Ω-m, respectively. The lower section of the profile shows low resistivity values of below 200 Ω-m. Comparison of the first and last profiles of the total measurement period shows an overall trend, i.e., the lower part of the profile has decreased resistivity from 14 October to 20 December. The very shallow part close to the soil surface shows a trend towards slightly higher resistivity values, particularly on the eastern side of the ERT transect.

The duplicate ERT measurements, i.e., immediately carrying out multiple resistivity measurements behind each other with assumed unchanged soil conditions, showed instrument errors of <3% for 9 November and <1% for 20 December with consistently low root mean square (RMS) values of 1.07 ± 0.07 and 0.99 ± 0.08 and least squares (LS) values of 0.29 ± 0.04 and 0.25 ± 0.04, respectively, for the inversion of ERT measurements. These uncertainties of the instrument measurement values are very small and are expected to have no or only minor impact on the signal of soil moisture patterns.

### 4.1. Moisture Profiles

**Figure 6:**
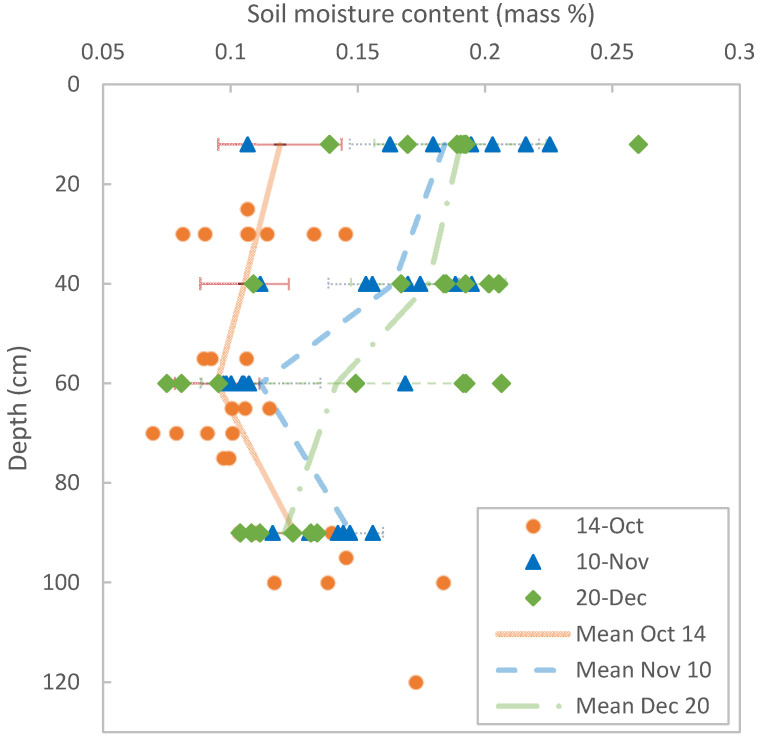
Soil-moisture measurements at the three dates, represented by circles, triangles, and diamonds. The lines represent the average soil moisture of the whole transect at that date. Average values for 14 October have been inter- and extrapolated for the sake of comparison with the other average values. The error bars represent the standard deviation of all measurements.

**Figure 7:**
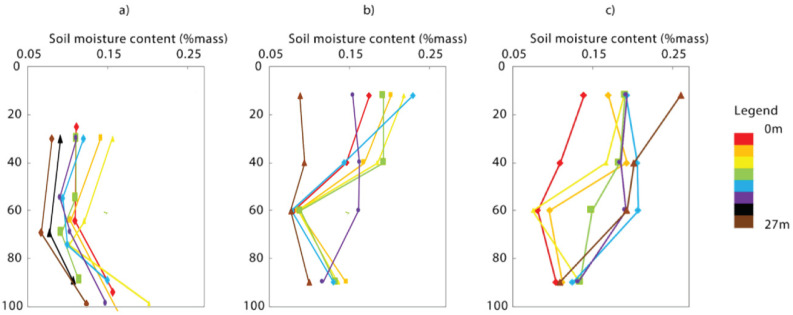
Individual soil moisture profiles taken on the ERT transect at different distances and at three dates: (**a**) at 14 October, (**b**) at 10 November, and (**c**) at 20 December.

The gravimetric soil moisture samples taken near the ERT transect on the three different dates to evaluate relation and moisture dynamics are presented in Table 1. During this period, the soil became progressively wetter from top to bottom in the three months of measurements, as is shown in Figure 6, where the gravimetric soil measurements are averaged along the ERT transect for the three sampling dates. Figure 7 shows the soil moisture increase as measured by the gravimetric method along the ERT transect (in colors where 0 m is west, and 27 m is east) for the three dates. Soil moisture is not uniformly distributed along the transect: in October the soil is wetter at the western part of the transect than at the eastern part, while in December this trend has reversed, with the eastern part of the transect being wetter (Figure 7). Closer to the groundwater, at the bottom of the auger pit, the profiles all approach the same value, regardless of timing or location along the transect.

**Figure 8:**
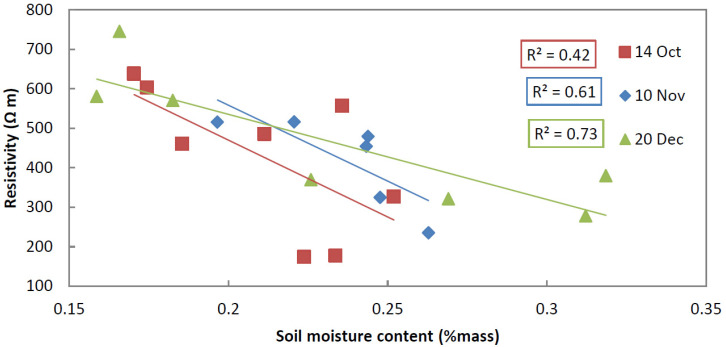
Correlation between soil moisture and resistivity at the depth of 90 cm indicated in Figure 5.

Figure 8 illustrates the correlation between measured gravimetric soil moisture and ERT measured resistivity, i.e., the closest pixel value, for a depth of 90 cm, as indicated by the dashed line in Figure 5, for the three dates and for all auger locations. To calculate the regression line and coefficient, we assumed a relationship based on Archie’s Law [33,34]. Archie’s law relates the (in situ) electrical conductivity of a material to its porosity and saturation. The resistivity of a rock is proportional to the resistivity of the dry material and the resistivity of the pore space. Archie’s law is valid in clean porous media with a non-conductive rock matrix, like pure sands, so without considerable amounts of clay [9]. Soil moisture values have been interpolated for non-sampled locations. Correlation for the three dates range from 0.43 to 0.73. The correlation between these two variables was best for a depth of 90 cm, where the soil consists of pure aeolian sand and where the variation of water content is large. Correlation at a depth of 60 cm are a bit lower and range between 0.43 and 0.61. Correlation at 90 cm depth is best for the samples collected on 20 December with an *R*^2^ of 0.73 (*p*-value of 0.014), followed by the samples collected on November 10th with a *R*^2^ of 0.65 (*p*-value of 0.053) and followed by the samples of 14 October having a *R*^2^ of 0.43 (*p*-value of 0.079). Correlations are reasonable, but ideally (n of samples) populations should be larger to increase significance.

### 4.2. Time-Lapse ERT

**Figure 9:**
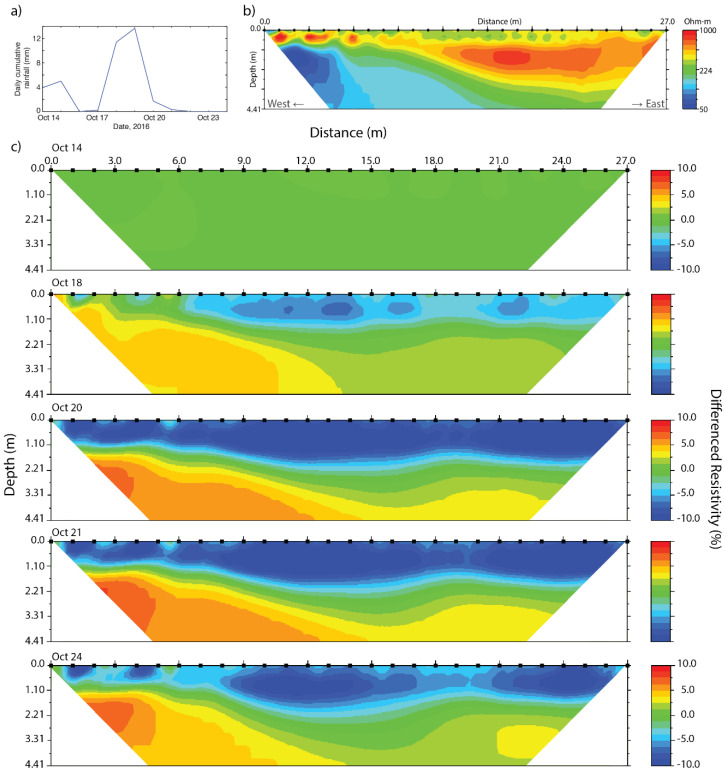
Time-lapse resistivity profiles, indicating soil moisture dynamics, with (**a**) rainfall from 14 October to 24 October, (**b**) the base profile of 14 October, and (**c**) the differenced profiles from 14 October to 24 October. Note the anomaly at the far left side of the profile (further discussed in the text).

**Figure 10:**
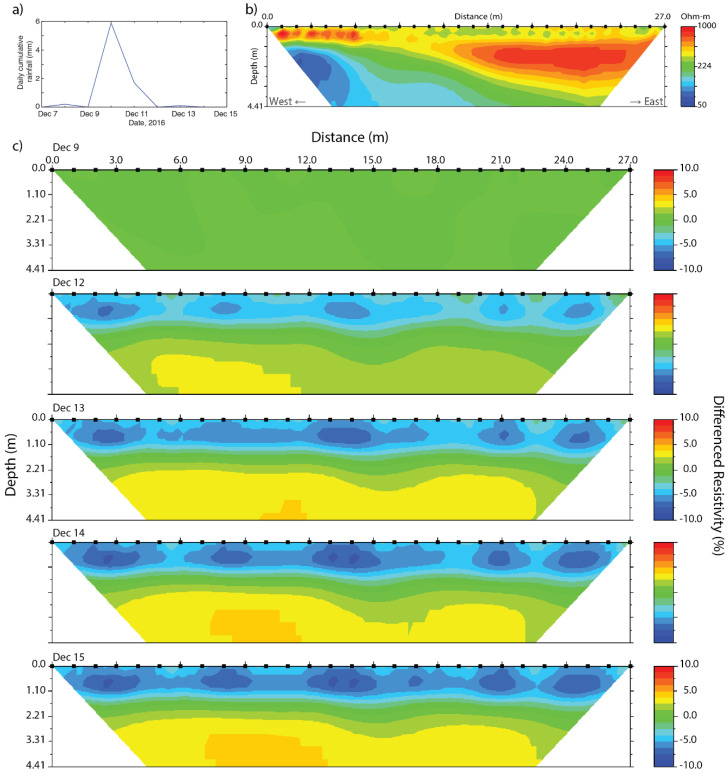
Time-lapse ERT resistivity profiles, illustrating moisture dynamics, with: (**a**) rainfall from 7 to 15 December, (**b**) the base profile of 9 December, and (**c**) the differenced profiles from 9 December to 15 December.

To discern trends and dynamics in soil moisture during precipitation events we used time-lapse ERT. This method uses a base profile and at least one profile obtained at a different time at the exact same location, resulting in the resistivity difference between the measurement moments. In this paper, the profiles obtained during two subsets of the data taken during two rainy periods have been used (Figure 9 and Figure 10). The effect of temperature changes on resistivity in the upper 25 cm layer is 8% in October and 2% in December, where observed resistivity changed up to 10%. The top layer of the difference profiles in October will therefore not be used for analysis, while in the difference profiles of December temperature changes may be neglected.

The first time-lapse ERT was taken from 14 to 24 October, using five ERT profiles (Figure 9). Prior to this period there was little precipitation and from 14 to 21 October, 36 mm of precipitation was measured. This has caused up to 15% decrease of resistivity at a depth range of 60–180 cm on the 20 October. The resistivity increases again during a period of no precipitation, but by the 24th it locally remains 10% lower than at the 14th. Below the observed groundwater table (at 1.80 m) there are also patterns present. The most prominent anomaly can be seen at transect meters 1 to 3 at the western side of the ERT transect, i.e., left in Figure 9c. Here, an increase of up to 12% in resistivity is observed at a depth range of 60–180 cm. The location of the increase concurs with the location of the oak tree.

The second time-lapse ERT was taken from 9 to 15 December (Figure 10). Before this period precipitation amounts were near monthly average (www.knmi.nl), with a total amount of 77 mm of precipitation in November versus 82 mm of average precipitation for this month. The last significant event was on 21 November, with 21 mm of precipitation. At 10 December a small precipitation event occurred, with 6 mm of rain. Accordingly, there is up to 7% decrease of resistivity in the top layer, which persists until at least five days after the main precipitation event without change. There is a more or less homogeneous increase in resistivity of the groundwater layer at 1.80 m depth and deeper.

## 5. Discussion

In this paper we assess the suitability of ERT to assess the spatial and temporal distribution of soil moisture along a transect of 27 m in a homogeneous soil profile. The setting of our experiment is in between an inside lab experiment with controlled conditions and a true field experiment where many environmental factors are difficult to regulate or to account for. Using time-lapse inversion of multiple resistivity measurements of the same transect we track the infiltration of precipitation into the soil, and fit experimental relations between ERT data and gravimetric soil moisture samples. We evaluate error characteristics of the ERT data itself, the inversion process, and differences in the spatial footprint of the sampling methods.

Time-lapse ERT shows clear trends in the unsaturated zone following precipitation events. The delay time of rain infiltration could not be determined in this experiment because the measurement frequency was below the typical time scale of this process. To extract trends from the time-lapse ERT that are more defined, it is advised to increase the frequency of measurements of resistivity which may only be feasible with an automated logging functionality in the ERT equipment. These types of problems of temporal under-sampling (sometimes referred to as aliasing) and long integration time of the measurement method resulting in motion blur, are known and described problems in literature, e.g., [35]. The proposed solution to reduce measurement time and reducing spatial resolution is not always feasible.

### 5.1. Uncertainty in the Gravimetric Moisture Reference Data

**Figure 11:**
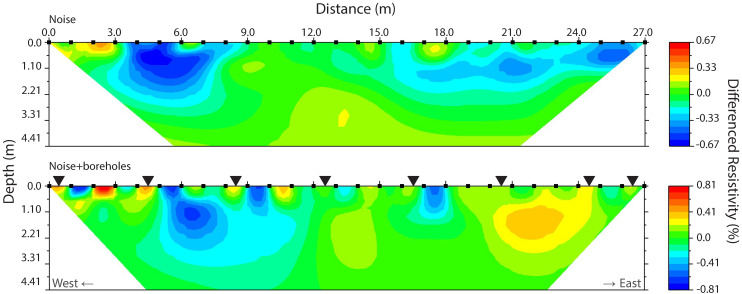
Comparison between regular noise (**top**) and noise after drilling boreholes for gravimetric sampling exactly located on the transect of two ERT measurements on 20 December (**below**). The black triangles in the lower transect indicate the location of the boreholes.

As a moisture reference for the ERT we use gravimetric moisture samples taken in boreholes by auger. Using boreholes was deemed to be the least destructive method for obtaining gravimetric soil moisture measurements. While carrying out time-lapse ERT measurements for evaluating soil moisture dynamics it is essential to let the transect unchanged, i.e., no gravimetric soil moisture sampling should be done at the exact location of the transect. As an extra precaution, during the experiment, it was decided to keep a distance of 4 m away from the ERT transect to place the boreholes but parallel to the ERT transect. After ending the experiment on 15 December, boreholes were placed on the ERT transect and one more ERT measurement was done to evaluate the effect of the boreholes along the ERT transect and vertical profile. As the soil profile, land cover, and topography of the site is very homogeneous, the effect of this distance on the correlation will be small. The difference in resistivity between the presence of boreholes in the profile and an uncompromised profile is lower than 1% as the measurements on 20 December show (Figure 11). The boreholes are however clearly recognizable. Therefore, the distance of 4 m parallel to the ERT transect is the best compromise of not disturbing the time-lapse measurement and collecting accurate gravimetric soil moisture values. The samples taken directly on top of the transect at the end of the experiment on 20 December do show slightly better correlation with resistivity values than those at 4 m from the transect (Figure 8).

Various other sources of error are identified during the gravimetric soil moisture measurements due to the method and because of the lithology. Utilizing boreholes makes it possible to mix in soil from the sides of the borehole with the sample taken at the bottom of the borehole. Furthermore, as the sand gets progressively wetter towards the ground water, the sand will not effortlessly remain in the sampler and some compaction is required to recover the sample. Significant correlation of 0.73 between resistivity and soil moisture is only present for samples of 20 December taken in pure sand and directly on top of the transect. Sandy substrate seems to be best suited for direct correlation of soil moisture with resistivity, provided that enough samples are taken.

### 5.2. Noise in the ERT Data

The ERT inversion profiles have low levels of noise. We based this on the duplicate measurements [34] done where we measured the same transect twice directly after each other (Figure 11 top) and low root mean square errors and LS values for the inversion indicating stable and consistent measurements. Figure 11 below illustrates the effect of the boreholes on the transect and the measurements. The disturbing effect of the auguring on the transect is significant and gravimetric sampling should be done at a distance of at least two or three meters away from the ERT transect. The ERT profiles had lower error levels towards the end of the experiment; where on 14 October duplicate measurements show noise levels of maximum 3% in resistivity variation, noise decreased to below 1% on November 11. The decrease in measurement noise may be caused by settling of the electrodes into the soil and increased wetter conditions in the topsoil, both resulting in decreased contact resistance between the electrodes and the soil. Our duplicates measurements and the resulting estimated noise should be interpreted with care, as [34,35] showed that the error estimate of using such stacking approach may be low compared to other more advanced error estimates based on reciprocal and short-term repeatability approaches. However, in our measurements these were all below 2% and similar to the uncertainty, or error, of the complete repeated survey.

### 5.3. Effects of Objects along the Field Transect

Given the low noise level and low repeat measurement error we consider the patterns in the ERT accurate. However, in the top 60 cm of the ERT profile measurements may be influenced by the presence of some clay in the upper part of the soil column resulting in a non-stationary relation between resistivity and soil-moisture content. In the homogeneous sand below the ground water patterns are observed that deviate from the expected homogeneous resistivity. The oak tree and the ditch with conductive surface water at the west end of the transect shown in Figure 1 may have an influence on the patterns. The preferential flow path through the roots of the tree might decrease the resistivity in this part of the soil seen in Figure 9 for the October time-lapse. In the December time-lapse of Figure 10 this pattern has disappeared, which may be an effect of seasonal changes in tree physiology or the general increase of soil moisture during the autumn months. Experiments by Cassiani et al., Mary et al. and Michot et al. [36,37,38] describe effects by roots on ERT in detail. The infiltrating surface water from the ditch with dissolved substances, and hence higher conductivity may also play a role in lowering soil resistivity. The difference in conductivity between groundwater and surface water was measured and yield 251 μS/cm for ground water and 720 μS/cm (both at 3 °C) for water in the ditch. The surface water with lower resistivity may have infiltrated in the soil near the western end of the transect causing the patterns left in Figure 5 and Figure 9, while only ground water with lower conductivity (and thus higher resistivity) is present in the other parts of the transect and profile. Therefore, the increased resistivity suggests that towards the end of the experiment ground water was replacing the surface water (that had infiltrated into the ground during summer) because of the hydrostatic pressure gradient from the ditch with surface water towards the east of the ERT transect. The low base values of resistivity in the western part also contribute to the large relative changes because small absolute values of deviation have a larger relative (percent change) effect on this section.

### 5.4. Relations with Other Studies and Outlook

In our experiment, in what we call in the introduction, the next best outside lab, we anticipated to obtain good results given the homogeneity of the soil profile and the controlled conditions with respect to, e.g., land cover and water table. However, using the results of our study, we are not capable of making similar claims of the suitability of ERT-measurements for soil moisture dynamics assessment as the laboratory studies of [11,12], i.e., environmental factors and noise remain playing disturbing roles in the measurements outside and are resulting in lower correlations and less accurate moisture estimates. Our homogenous profile was expected to overcome problems with variable soil properties and to exclude the necessary use of pedotransfer functions as discussed by [24,27,38]. It should be noted that pedotransfer functions are in general necessary to convert ERT measurements into quantitative soil moisture information [27,37]. In our experiment it was feasible to partly explain soil moisture spatial patterns after rainfall events, i.e., the development of the wetting front, and to correlate them to gravimetric moisture measurements. Quantitative relations between ERT measurements and soil moisture are modest and are similar to many other studies [13,14,20,27]. This modest correlation can partly be explained by different conductivity properties of rainwater and surface water in the ditch versus groundwater. Furthermore, the wetting front is only feasible if ERT measurements are carried out at an hourly or half-day basis and our long-time steps failed to capture rainfall infiltration. Interesting approaches for further study are performing ERT measurements at much shorter time steps and to apply spatial interpolation algorithms to spatially smooth ERT data before correlating them to gravimetric soil moisture measurements as suggested by [16,20].

## 6. Conclusions

A field experiment was carried out to use electric resistivity tomography (ERT) to study the dynamics of soil moisture patterns after rainfall for a homogeneous soil on a study site in the central part of The Netherlands. Using repeated ERT measurements of this homogeneous soil transect we found clear infiltration patterns and increasing soil moisture content patterns in the unsaturated zone following two major precipitation events. It was not possible to accurately follow the rainfall infiltration front at the measuring intervals used. More frequent ERT measurements are required to capture in detail the infiltration front.

Gravimetric soil moisture measurements were carried out for three dates at the start, the middle, and the end of the experiment along the ERT transect and to a depth of 1.20 m. Gravimetric soil moisture content was correlated with resistivity values for the three dates and for various soil depth. Correlations ranged from 0.43 for October until 0.73 for December and best value were found for a depth of 90 cm.

The measurements also showed the effects of changes in ground water conductivity from autumn to winter and some effects of preferential flow paths along tree roots. The intrinsic noise of the ERT values was evaluated by duplicate measurements, the resulting low root mean square and least square values indicate low noise values. However, some artefacts in the ERT profiles are difficult to explain and could be a remnant of the inversion software. Although the selected site consists of a homogeneous grass field and has a fairly homogenous sandy soil transect, an oak tree and a ditch with surface water at the west end of the ERT line had notable impact on the resistivity patterns. Even in this relatively easy and controlled field site, it is not straightforward to find strong relations between ERT measurements and soil moisture, and it remains difficult to obtain the accuracies of laboratory studies using ERT.

While the effects of clay presence in the soil and possible changes in ground water conductivity must be carefully considered, ERT is found to show a moderate correlation with soil moisture. ERT allows for detailed spatial measurement of soil moisture estimates and the patterns that result from precipitation and evapotranspiration, and it is especially suited for inaccessible soil and detailed spatial analyses, where probe or sample-based methods fall short.

## Figures and Tables

**Table 1:** A summary of the soil samples gathered (see also Figure 1).

Date	Boreholes	Samples	Maximum Depth (cm)	Distance from ERT Setup (m)
14 October	8	28	120	4
10 November	7	28	90	4
20 December	7	28	90	0
